# Influence of Sodium Hydroxide Treatment on *Typha domingensis* Fibers for Geotextile Manufacturing

**DOI:** 10.1021/acsomega.4c05602

**Published:** 2024-12-16

**Authors:** Francisco Sandro Rodrigues Holanda, Luiz Diego Vidal Santos, Jeangela Carla Rodrigues Melo, Alceu Pedrotti, Eliana Midori Sussuchi, Sandro Griza, Renisson Neponuceno de Araújo Filho, Brenno Lima Nascimento

**Affiliations:** † Department of Agronomy Engineering Rosa Elze, Universidade Federal de Sergipe-UFS, São Cristóvão 49100-000, Sergipe, Brasil; ‡ Postgraduate Study in in Territorial Planning, Department of Human Sciences and Philosophy, Universidade Estadual de Feira de Santana - UEFS, Avenida Transnordestina, Novo Horizonte, Feira de Santana 44036-900, Bahia, Brazil; § Department of Chemistry, Universidade Federal de Sergipe-UFS, Rosa Elze, São Cristóvão 49100-000, Sergipe, Brasil; ∥ Postgraduate Study in Materials Science and Engineering, Universidade Federal de Sergipe-UFS, Rosa Elze, São Cristóvão - SE 49100-000, Brasil; ⊥ Department of Rural Technology, Universidade Federal Rural de Pernambuco-UFRPE, Rua Dom Manuel de Medeiros, s/n Dois Irmãos, 52171-900 Recife-PE, Brasil

## Abstract

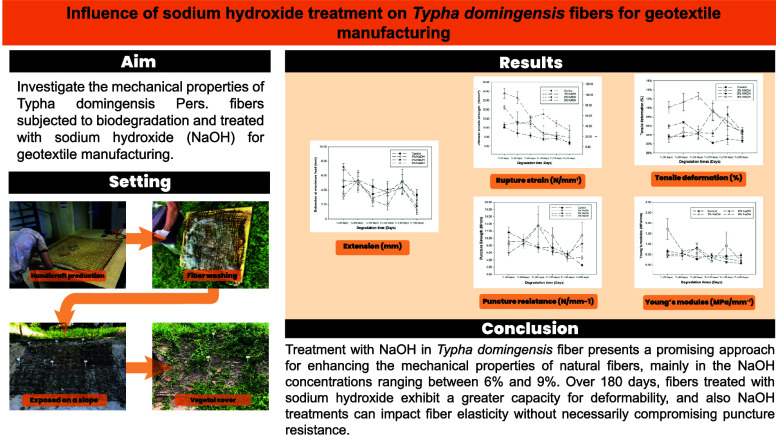

The conservation of soil, a finite natural resource, demands effective measures. Within this context, the instability of soil masses on steep slopes poses significant risks to human life and environmental infrastructure, highlighting the need for developing erosion control strategies rooted in soil bioengineering principles. The objective of this study was to investigate the mechanical properties of *Typha domingensis* fibers subjected to biodegradation and treated with sodium hydroxide (NaOH) for geotextile manufacturing. Experimental slopes were employed to mimic natural environmental degradation conditions. The *Typha domingensis* fibers underwent treatment with alkaline NaOH solutions at concentrations of 3, 6, and 9% and were exposed for 180 days. Samples were collected every 30 days to evaluate the degradation process and performance under these conditions. These fibers exhibited resilience against field degradation over a period exceeding 180 days, demonstrating sustained effectiveness. Despite an initial reduction in strength compared to untreated control fibers, the treated fibers displayed enduring stability throughout the experimentation. This suggests that 6% NaOH concentration may yield higher tensile strength, thus positioning it as the optimal choice for the production of biodegradable geotextiles derived from *Typha domingensis* fibers.

## Introduction

Soil degradation presents a significant environmental challenge, impacting soil ecosystems, agricultural lands, and water resources while also contributing to siltation in rivers and dams.^1^ This degradation primarily stems from soil weathering, a vital process in landscape formation and alteration.^2^

Erosion occurs through surface runoff driven by rainfall, inflicting substantial damage due to the erosive force of water.^3^ Such runoff can induce laminar erosion, characterized by subtle sediment and particle transport, rendering it less conspicuous yet more insidious.^4^ Anthropogenic activities notably disrupt the natural balance between soil formation and erosion, potentially intensifying erosive processes. It is crucial to acknowledge that poorly managed practices can accelerate erosion rates,^4^ leading to substantial losses of soil, nutrients, water, and organic matter, thereby diminishing soil productivity.^5^ To address this degradation and curtail further erosion, researchers have explored traditional soil restoration techniques involving physicochemical processes.

In recent years, soil bioengineering has emerged as a sustainable method for soil stabilization aimed at restoration.^6^ These biotechnical approaches amalgamate vegetation with inert materials to stabilize soil and protect against erosion, providing a feasible and sustainable alternative. They serve as supplementary or even alternative measures to conventional civil engineering practices. The increasing pursuit of environmentally friendly alternatives and growing concerns about environmental pollution have highlighted the importance of biodegradable geotextiles grounded in natural bioengineering^7^

Geotextiles find extensive application in both engineering and agriculture, particularly for erosion control purposes. Their efficacy lies in promoting vegetative growth and soil drainage, considering the type of fibers employed and their integration into the soil matrix.^8^ Globally, approximately 1.5 billion square meters of geotextiles are utilized annually, with market projections estimating a value of US$10 billion by 2027, having grown at a compound annual growth rate (CAGR) of 9.6% during 2022–2027.^9^ Their appeal stems from their cost-effectiveness and versatility, fulfilling diverse needs ranging from mechanical properties to hydraulic and biological functions.^10^

The escalating utilization of petroleum-derived polymers in geotextile manufacturing, a significant contributor to solid waste and environmental degradation,^11^ has prompted a search for alternatives based on natural materials, such as plant fibers. Predominantly manufactured from nondegradable substances like polypropylene, polyethylene, and polyethylene terephthalate, most geotextiles pose environmental pollution risks. Therefore, there is an increasing interest in more sustainable alternatives, such as natural fibers or biodegradable polymers, which could potentially supplant up to 50% of nonbiodegradable materials in engineering applications.^12^ This transition underscores a burgeoning preference for sustainable options leveraging natural fibers, providing not only environmental advantages but also competitive mechanical properties.

Within the realm of natural fiber-based geotextiles, *Typha domingensis* is considered a particularly promising candidate.^13^ Naturally growing in swampy ecosystems, this plant yields fibers renowned for their robust tensile strength and durability.

*Typha domingensis* belongs to the division of *Angiosperms*, class *Monocotyledons*, order *Pandanales*, and family *Typhaceae.*(14) Typically found in marshes and along lake edges, the plant features a partially creeping and partially erect stem, reaching heights of up to 2.50 m.^15^ It boasts long, linear leaves and dark brown flower spikes. Abundant rhizomes and seeds enable rapid propagation.^16^ The leaves, rich in pure cellulose, are utilized for crafting mats and baskets.^17^ With a nearly cosmopolitan distribution, particularly in the northern hemisphere, there are 10 to 15 species of *Typha*, one or two of which are native to Brazil.^18^ It is regarded as an invasive species and traditionally managed in various regions, with its components harvested for crafting household tools and crafts by farmers and riverside dwellers.^19^

The *Typha* genus plays a significant role in phytoremediation, utilizing species such as *Typha angustifolia*, *Typha domingensis*, and *Typha latifolia* to extract heavy metals from water, soil, and sediments in both natural and artificial wetlands.^20^ Utilizing *Typha domingensis* not only aligns with sustainable management practices but also harnesses the innate properties of natural materials often neglected in industrial applications.^21^ These fibers possess a distinctive cellular structure reinforced with lignin, hemicellulose, and cellulose, providing the mechanical strength and flexibility required for demanding stress applications in geotechnical engineering.^22^

*Typha domingensis* proves particularly advantageous in endeavors related to soil erosion control, slope stabilization, and road structure reinforcement.^23^ Its integration into soil bioengineering processes enhances soil structure and promotes vegetation growth, rendering it an exceptional choice for ecological restoration initiatives.^24^ The development of geotextiles derived from *Typha* fibers not only promotes environmental sustainability but also fosters innovative engineering solutions that harmonize technological advancements with natural ecological processes.

Despite the environmental and sustainable advantages of natural fibers like *Typha domingensis*, they possess limitations in terms of durability compared to their synthetic counterparts. Natural fibers inherently undergo faster biodegradation due to their organic composition.^25^ While biodegradation offers benefits in reducing environmental impact and facilitating ecological cycles, it also means that these fibers may not provide the long-term structural stability required in certain geotechnical applications^26^

The biodegradable behavior of natural fibers results in a decline in tensile strength over time, potentially jeopardizing the effectiveness of geotextiles in critical infrastructure projects unless appropriately managed or treated.^27^ Consequently, while *Typha* fibers excel in projects with shorter lifespans or where environmental conservation is paramount, such as agricultural initiatives, their use demands careful consideration in scenarios requiring prolonged durability.

The degradation of the *Typha* fiber structure occurs through two primary mechanisms: surface erosion and radial penetration.^28^ Surface erosion entails the gradual breakdown of the outer layer, progressing to the cortical layer.^29^ Degradation initiates in the intercellular membrane of the cuticle. As the membrane complex deteriorates, individual cells gradually detach from each other. Concurrently, enzymes penetrate the cystine-poor cuticular sublayer and the endocuticle.^30^

The most resistant layers, namely the exocuticle and the endocuticle, undergo gradual degradation. After the cuticle breaks down, biodegradation begins within the cortical layer by affecting the intercellular space, leading to the separation of spindle-shaped cortical cells.^31^ Subsequently, biodegradation penetrates the cell interior, targeting the fiber situated within the macrofibrils. Consequently, individual microfibril bundles are disassembled and eventually decomposed.

The second mechanism, radial penetration, involves the infiltration of fungal hyphae perpendicular to the fiber surface. These penetrating structures act akin to drills, creating small-diameter tunnels through the cuticular layer. Once tunnels are drilled, enzymes diffuse laterally within the fibers, gradually digesting the cortical layer. Despite the intact cuticle, large cavities form within the cortical layer, ultimately resulting in the collapse of larger internal cavities.

Both mechanisms, surface erosion and radial penetration, typically occur concomitantly.^32^ The rate and mechanism of biodegradation are significantly influenced by factors including the fiber’s condition, product structure, and various environmental variables such as burial conditions, geographic location, exposure to ultraviolet light, soil composition, temperature, and humidity. Studies on archeological textiles have demonstrated the profound impact of these conditions on biodegradation.^33^

However, natural fibers’ biodegradation can be slowed down with chemical treatments and protective coatings. There are several methods to improve the physical or chemical properties of natural fibers, including blending them with thermoplastic polymers.^34^ One widely used method is alkaline treatment, also referred to as mercerization, because it is cost-effective. This method involves treating fibers with concentrated sodium hydroxide (NaOH) solution.^35^ In a study by,^36^ authors compared banana fibers that were untreated with those treated with a 5% NaOH solution for 4 h using electron micrographs. They noticed modifications on the fiber texture, which they attributed to the ionization of hydroxyl groups caused by the addition of sodium hydroxide solution.

Alkaline treatment using NaOH is known to significantly improve the properties of natural fibers, making them more suitable for geotextile applications.^37,38^ For instance, NaOH treatment enhances the removal of impurities and increases fiber-matrix adhesion, leading to improved mechanical performance of natural fiber composites.^39^ This process has been shown to enhance the tensile strength of fibers such as kenaf and hemp, both under dry and wet conditions, by removing lignin and hemicellulose components that contribute to fiber degradation.^40^ Additionally, studies have demonstrated that NaOH treatment alters the surface morphology of fibers, making them rougher, which improves the bonding with polymer matrices and enhances the overall composite properties.^41^

Thus, the primary advantage of *Typha* fibers in geotextile applications lies in their abundance and renewability, positioning them as a sustainable and cost-effective raw material. These fibers exhibit notable tensile strength and water retention capacity, which contribute to enhanced durability when compared to other natural fiber materials.^25,42^ Furthermore, the fibrous structure of *Typha domingensis* provides excellent permeability, making these geotextiles particularly effective for erosion control and soil reinforcement.^43^ Unlike synthetic geotextiles, natural geotextiles made from *Typha domingensis* do not release microplastics into the environment and do not interfere with agricultural activities, thereby avoiding issues such as clogging of agricultural machinery and equipment.^44^

Therefore, to enhance understanding of the durability of geotextiles made from natural fibers, this study aims to investigate the mechanical properties of *Typha domingensis* fibers subjected to biodegradation and treated with sodium hydroxide (NaOH) for geotextile production.

## Materials and Methods

### Plant Collection and Sample Processing

The selection of *Typha domingensis* Pers. was based on its mechanical properties as well as the cellulose and lignin content reported in the literature for each species. Leaves and shoots were collected from plants in two municipalities along the Lower São Francisco River in northeastern Brazil between 2020 and 2022.

This plant typically grows between 2 and 3 m in height and is characterized by brown flowers surrounded by green leaves.^45^ Its long, flat leaves have a cylindrical stem and are commonly utilized for constructing roofs, baskets, and mats. With a high cellulose content similar to other plants like *Boehmeria nivea* Gaud and *Agave sisalana* Pierre, *Typha domingensis* exhibits robustness. Moreover, the presence of lignin in *Typha domingensis* fibers contributes to their biodegradation resistance.^46^

*Typha domingensis* plays a vital role in wetland ecosystems by serving as a natural filter, removing excess nutrients and pollutants from water.^47^ Recognized for its broad range of uses and ecological benefits, *Typha domingensis* underwent formal identification by the Botany Laboratory of the Institute of Biology at the Universidade Federal da Bahia in northeastern Brazil. Plant samples were archived in the Herbarium of the Universidade Federal de Sergipe, Brazil. The collection of wild plant specimens in Brazil requires mandatory licenses and adherence to standard procedures recommended by the National System for the Management of Genetic Heritage and Related Traditional Knowledge (SisGen).^48^ Therefore, collection activities were conducted under the SisGen registration code A2B3842.

### Implementation of Field Experiments

The geotextiles utilized in this investigation were previously fabricated by the Erosion and Sedimentation Laboratory of the Federal University of Sergipe, employing *Typha* fibers. The production of geotextile prototypes involved four primary stages: (1) cutting and drying of the fibers; (2) grouping; (3) geotextile manufacturing; and (4) chemical application of NaOH. Twelve samples were used for each treatment and analysis period to ensure statistical reliability and robust evaluation of the degradation and performance characteristics.

The extraction of plant material was conducted meticulously, utilizing smooth-bladed tools to prevent fiber damage. Primary incisions in *Typha* fibers were executed above the plant roots to allow regrowth. After cutting, the fibers underwent a drying process in a shaded, dry environment, spanning approximately 8 days. Between the drying phase and the beginning of geotextile fabrication, the fibers were adeptly bound with ropes and arranged in bundles weighing approximately 5 kg.

**Figure 1:**
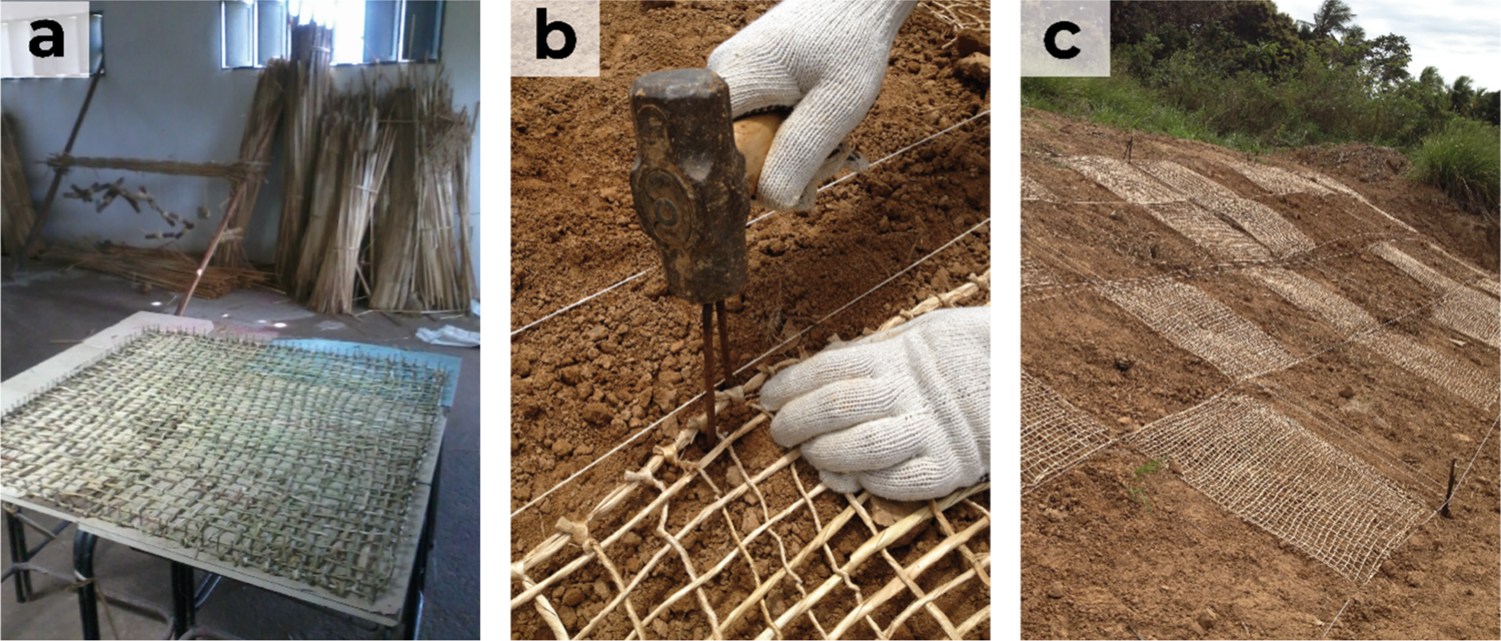
(a) Geotextiles manufactured with *Typha domingensis* fibers, coated with protective resin; (b) Geotextiles installed on the ground and (c) Geotextiles exposed on slopes.

Following drying, the fibers were meticulously intertwined until they formed a cord with a diameter of approximately 6 mm. This cord, placed on a substrate, constituted the biaxial weave of the geotextile. Arranged in a checkerboard pattern with squares measuring 25 cm^2^ spaced apart, the total mesh size amounted to 1.20 m^2^ (Figure 1).

The geotextiles manufactured with *Typha domingensis* underwent treatment with alkaline sodium hydroxide (NaOH). The blankets were immersed in NaOH solutions with concentrations of 3, 6, and 9% for 24 h.^49^ For every 60 L of water, 1800 g of NaOH (0.75 mol/L) were used for a concentration of 3%, 3600 g of NaOH (1.5 mol/L) for a concentration of 6%, and 5400 g of NaOH (2.25 mol/L) for a concentration of 9%. Subsequently, the treated geotextiles were rinsed with running water and left to air-dry at room temperature.

The objective of the alkaline treatment process with NaOH solution was to reduce permeability and delay degradation, thus enhancing the material’s resistance to climatic factors. To ensure comprehensive coverage, waterproofing resin was applied to both sides of the geotextiles. The geotextiles underwent the following treatments: (a) geotextile without waterproofing resin (control); (b) geotextile treated with a single layer of waterproofing resin at a concentration of 0.324 mg/mL; (c) geotextiles treated with two layers of waterproofing resin at a concentration of 0.648 mg/mL.

To assess biodegradation behaviors, five samples were collected from the central region of each geotextile during scheduled collections, with each fiber measuring 90 mm in length by 10 mm in diameter, over a maximum exposure period of 180 days (six months). The exposure duration aimed to evaluate the durability of biodegradable geotextiles, representing the maximum effective durability period of the geotextile in the field. For many geotextile applications, longevity and durability are crucial factors in fulfilling their role in soil reinforcement and protection.^50^ These samples were chosen from geotextiles that maintained their integrity during field exposure. The collection process encompassed four distinct time intervals: T_0_ (0 days), T_1_ (30 days), T_2_ (60 days), T_3_ (90 days), T_4_ (120 days), T_5_ (150 days), and T_6_ (180 days).

### Resistance Tests

To conduct tensile strength tests on the manufactured and treated geotextiles, samples that had undergone 180 days of degradation in the field, situated on the slope of the experimental area, were collected and subjected to mechanical resistance testing procedures.

The mechanical characteristics evaluated pertain to the requirements of geotextiles in stabilizing slopes, where they encounter traction, compression, and flexion forces, among others. Through these tests, the main parameters of mechanical resistance were observed, obtained from stress and strain curves at rupture as well as stiffness measurements.

**Figure 2:**
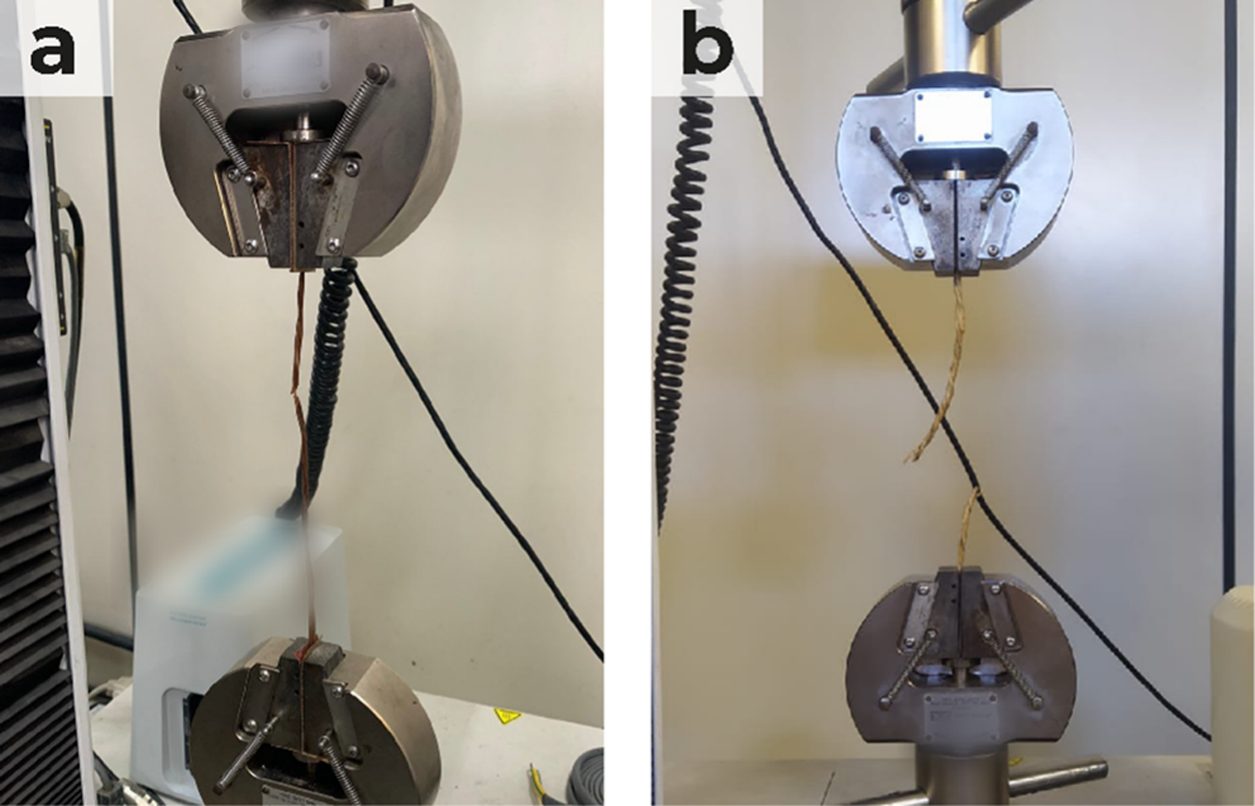
(a) Mechanical tests of *Typha domingensis* fibers subjected to chemical treatment with NaOH and (b) gripping device used in the equipment.

Tensile strength tests were conducted at the Laboratory of Materials Engineering at the Federal University of Sergipe, utilizing an EMIC Model DL universal testing machine (Figure 2) with a maximum capacity of 300 kN and a distance of 100 mm between the claws. To secure the geotextiles on the universal testing machine, steel claws were fabricated with rough or abrasive material on the inner faces for better adherence, thus ensuring proper fixation of the blankets and preventing displacement during force application.

Punch resistance tests were performed using Instron 3385 H universal testing equipment, at the same Laboratory. These tests followed the specifications of NBR ISO 10319 Geotextiles—Determination of Resistance to Static Punching—CBR Type Piston Test,^51^ differing only in the sample size, which conformed to specifications for Mini-CBR tests.

The samples were cut to a length of 30 cm and secured between the rings of the cylindrical support with an internal diameter of 50 mm. The punch was then moved downward at a speed of 50 mm/min, while the machine recorded the punching force versus penetration. Using these curves, the punch resistance and maximum penetration were calculated.

### Statistical Analysis

To assess the temporal progression of biodegradation across various treatments and fibers within the designated interval (from T_1_–first day to T_6_–180 days), a factorial analysis of variance (ANOVA) was conducted, considering multiple measurements. The primary aim of this analysis was to track the activity of biodegradation agents over time and investigate the impact of waterproofing resin application. The normality of data distribution was evaluated using the Kolmogorov–Smirnov (KS) and Shapiro-Wilk (SW) residual tests,^52,53^ while Levene’s test was employed to assess the assumption of homogeneity of variance.^54^

Discrepancies between initial natural degradability and degradability after 180 days of exposure were examined using the Bonferroni test, with a significance level of 5%. Effect sizes were calculated using Eta squared (η^2^) and Cohen’s d for the mean difference test (Δ*M*).^55^ These effect sizes quantify the strength of the relationship between an independent variable (IV) and a dependent variable (DV). In this study, the effect size obtained for DV indicates the impact of geotextile treatment compared to the control treatments (T_1_ and untreated with resin).

To increase result reliability and account for deviations from normality in sample distribution as well as differences in group sizes, bootstrapping procedures were implemented. These procedures help mitigate potential biases and increase the robustness of conclusions.

## Results and Discussion

### Maximum Tensile Strength

The results of the maximum tensile strength tests provide a comparison between the geotextile’s strength and its natural degradation in the field. Assessment of normality in the residuals of maximum tensile strength revealed that only the 30-day treatment with 0% NaOH exhibited a normal distribution (KS = 0.113, *p* < 0.200; SW = 0.997, *p* < 0.911), which is relevant for geotextile analysis as it ensures that the data align with the assumption of non-normality, justifying the utilization of bootstrapped data. Moreover, Levene’s test confirmed the nonhomogeneity of variance between groups, an important assumption for ANOVA, as indicated by the nonsignificant test results (*p* < 0.05), signifying that the variability within each group was similar under experimental conditions. Therefore, the assumption of homogeneity of variances was satisfied, bolstering the reliability of the ANOVA results.

**Figure 3:**
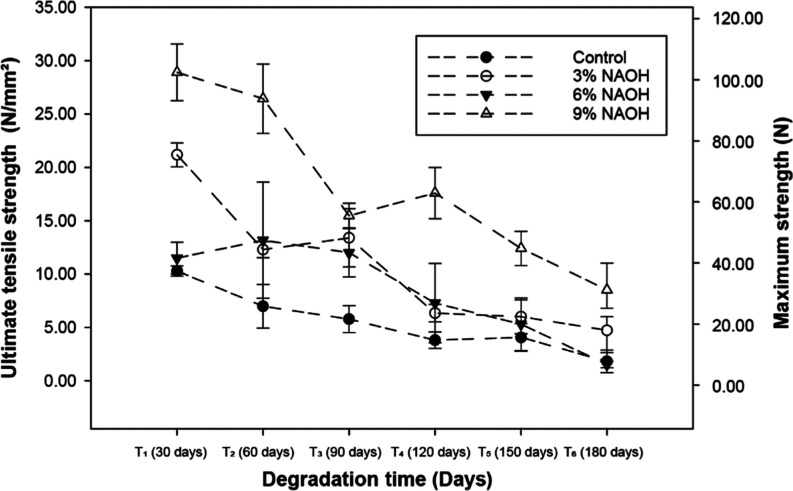
Ultimate tensile strength (N/mm^2^) and maximum strength (N) at *Typha domingensis* fiber rupture considering degradation time in the field.

Figure 3 illustrates the maximum tensile strength data of *Typha domingensis* fibers considering the degradation time in the field. The comprehensive ANOVA analysis performed in this study on the tensile strength of *Typha domingensis* fibers throughout a 180-day experimental period confirms these findings. The fibers exhibit statistically significant differences, with a substantial effect size (*F* (3, 71) = 35.564, *p* < 0.001; η^2^ = 0.835), signifying that the variations in tensile strength observed throughout the field experimental period were not arbitrary but rather the outcome of systematic factors directly impacting the material’s performance.

The decline in tensile strength over time highlights the influence of environmental variables on geotextile degradation. As degradation progresses, there is a gradual reduction in tensile strength, suggesting that environmental factors such as sunlight exposure, saprophytic microorganism actions, humidity, and temperature fluctuations play a significant role in deteriorating the mechanical properties of the fiber.^56−58^

Furthermore, the substantial effect size (η^2^ = 0.835) resulting from the analysis underscores the practical significance of these findings. This large effect size suggests that the decrease in tensile strength has significant implications for geotextile performance and longevity. Works such as Khalid et al.,^59^ and Vishnudas et al.,^60^ have emphasized the necessity of treating natural fibers with protective waterproofing measures to enhance their mechanical properties and moisture resistance in soil bioengineering applications.

The treatment of natural fibers with NaOH has become a common practice to enhance their physical and chemical properties, rendering them more suitable for various industrial applications, including the production of biodegradable geotextiles, which mitigate both superficial and radial penetration forms of biodegradation.

The alkaline treatment with NaOH acts on *Typha domingensis* fibers by modifying their internal structure, primarily through the removal of lignin, hemicellulose, and other impurities. This process exposes more cellulose, a component that provides greater strength and stability.^61^ Additionally, this treatment not only cleanses the fibers but also alters their morphology and crystallinity, resulting in fibers with rougher and more porous surfaces.^62,65^ These characteristics are crucial for geotextile manufacturing as they improve the adhesion of fibers with polymer matrices or other materials, thereby increasing the mechanical resistance of the final product.^63,64^ Similar behavior was also observed by 58 using bamboo fibers (*Bambusa vulgaris* vittata) as a starting compound.

Regarding tensile strength, pairwise comparisons revealed statistically significant differences in mean M (IJ) values for all treatments during the 180-day exposure period (Table 1). At the beginning of the experiment, it was observed that the 3% NaOH treatment led to an increase in maximum tensile strength compared to the control treatment, with Δ*M* = 10.903 N/mm^2^, indicating a statistically significant difference (*p* = 0.001).

**Table 1:** Maximum Fiber Tensile Strength at the Beginning of the Experiment (30 Days) and after 180 Days of Exposure to Natural Degradation in the Field

				95% CI for mean difference		
exposure time	treatment		mean difference Δ*M* (I–J)	bottom	highest	sig. bonf
30 days	control	3% NaOH	–10.903^a^	–19.48	–2.326	0.001
		6% NaOH	–1.207	–9.784	7.370	0.494
		9% NaOH	–18.626^a^	–27.203	–10.049	0.001
	3% NaOH	6% NaOH	9.696^a^	1.119	18.274	0.001
		9% NaOH	–7.722^a^	–16.299	0.855	0.010
	6% NaOH	9% NaOH	–17.419^a^	–25.996	–8.842	0.001
180 days	control	3% NaOH	–2.906	–11.483	5.671	0.251
		6% NaOH	0.282	–8.295	8.860	0.848
		9% NaOH	–6.704^a^	–15.281	1.873	0.024
	3% NaOH	6% NaOH	3.188	–5.389	11.765	0.137
		9% NaOH	–3.798	–12.375	4.779	0.283
	6% NaOH	9% NaOH	–6.987^a^	–15.564	1.590	0.013

a The mean difference is significant at a level of 0.05.

On the contrary, the use of 6% NaOH did not produce a significant alteration in the mechanical properties, showing only a marginal difference of Δ*M* = 1.207 N/mm^2^. However, employing a higher concentration of 9% NaOH yielded a greater increase in strength compared to the 6% NaOH concentration, with a mean difference of Δ*M* = 18.626 N/mm^2^ (*p* < 0.001), indicating a substantial enhancement in fiber strength with NaOH treatment. This phenomenon can be attributed to the improvement in the interfacial resistance of the fiber matrix following NaOH treatment, resulting in fewer voids that serve as sites for crack initiation in the composites. Consequently, the reduction in crack initiation sites in plant fibers leads to an improved impact resistance of the composites.

Furthermore, research by Khan et al.,^36^ demonstrated that banana (*Musa* spp.) fibers treated with NaOH exhibited significant improvements in all mechanical properties, particularly in tensile and compressive strength, which aligns with the findings presented in Table 1, wherein the application of 9% NaOH led to a notable increase in maximum resistance even at the onset of the experiment (30 days).

After the longest fiber exposure period (180 days), Table 1 presents data regarding the treatment with 9% NaOH at the conclusion of the field experiment, revealing statistically significant differences in tensile strength compared to control samples (Δ*M* = −6.704 N/mm^2^, *p* = 0.024, 95%BCa = −15.281 to −1.873), albeit with weak distinctions between the 6 and 9% NaOH concentrations (Δ*M* = −6.987 N/mm^2^, *p* = 0.013, 95%BCa = −15.564 to 1.590). Other treatments did not exhibit statistically significant differences at the experiment’s conclusion. However, Karthikeyan et al.,^66^ found that applying NaOH concentrations up to 10% to natural fibers enhances the tensile strength of composites, with the optimal concentration being 4% for coconut (*Cocos nucifera*) fibers. Nevertheless, concentrations exceeding 10% in NaOH led to a decrease in tensile strength. This observation aligns with the outcomes of the present study.

### Tensile Deformation

The tensile behavior of natural fibers from *Typha domingensis* treated with NaOH provide valuable data concerning the material’s performance under various treatment conditions and temporal exposures. Analysis of variance (ANOVA) reveals a substantial variation in geotextile deformation due to NaOH treatment, supported by a small effect size (η^2^ = 0.318), indicating that 31.8% of the deformation variability can be attributed to the different NaOH treatments applied. The F value of 12.986 and a *p*-value below 0.001 confirm the statistical significance of these differences. However, exposure to biodegradation, representing the exposure time, did not significantly impact deformation (*F* = 1.406, *p* = 0.239), accompanied by a small effect size (η^2^ = 0.057). This suggests that exposure time alone does not primarily influence material deformation during field testing.

The interaction between NaOH and deformation time was also relevant, with an *F* value of 1.906 and a *p*-value of 0.046, indicating that the effectiveness of NaOH treatments varies over time. This interaction accounts for 23.3% of the observed variability in deformation (η^2^ = 0.233), emphasizing the material’s complex response under different treatment and time conditions. Thus, the ANOVA data for deformation confirm that NaOH treatment significantly affects geotextile deformation, with this effect being modulated by exposure time.

**Figure 4:**
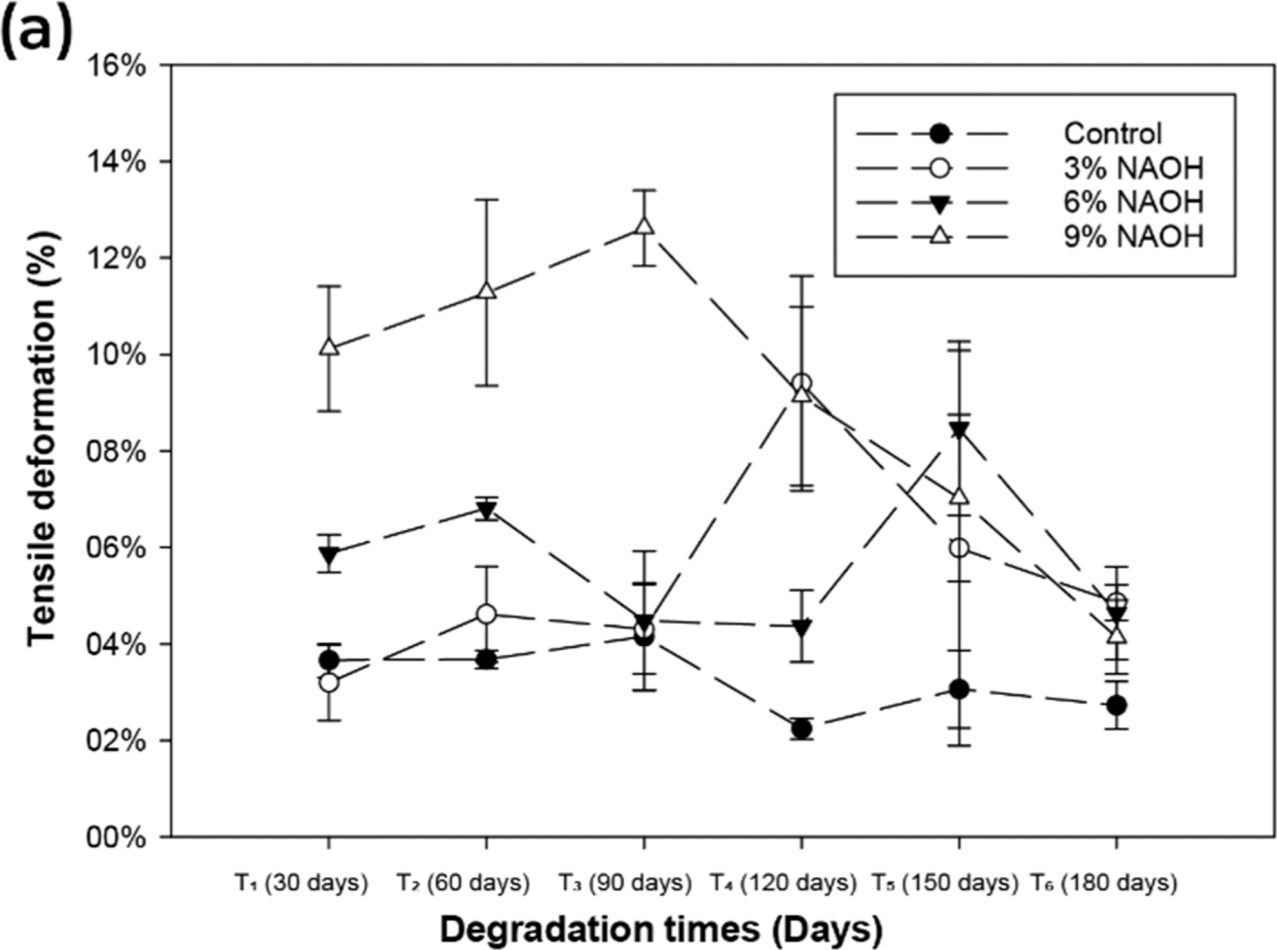
Tensile deformation (%) upon rupture of the *Typha domingensis* fiber considering degradation time in the field.

Figure 4 illustrates the data for tensile deformation at rupture of *Typha domingensis* fiber considering degradation time in the field. Regarding deformation, paired comparisons of the data during the field experiment reveal that at the experiment’s onset (30 days), the 3% NaOH treatment exhibited no significant difference in deformation compared to the control (*M* = 0.5%, *p* = 0.617, 95% BCa = −0.01 to 0.2%), suggesting minimal or no change. In contrast, the 9% NaOH treatment demonstrated a notable reduction in strain (Δ*M* = −6.2%, *p* < 0.001, 95%BCa = −8.3 to −3.8%), indicating a potential progressive increase in fiber stiffness.

The interaction between the analyzed variables indicates that differences in deformation result not only from NaOH levels but also from the duration of field exposure (Table 2). Over 180 days, the deformation data reveal that fibers treated with sodium hydroxide exhibit a greater capacity for deformability, notably higher with 3% NaOH compared to the control treatment (Δ*M* = −2.1%, *p* = 0.002, 95%BCa = −3.2 to −0.6%).

**Table 2:** Tensile Deformation of the Fibers at the Beginning of the Experiment (30 Days) and after 180 Days of Exposure to Natural Degradation in the Field^a^

				95% CI for mean difference		
exposure time	treatment		mean difference Δ*M* (IJ), %	bottom, %	highest, %	sig. bonf
30 days	control	3% NaOH	0.5	– 1.2	2.2	0.617
		6% NaOH	– 2.2	– 3.2	– 1.0	0.001
		9% NaOH	– 6.3	– 8.3	– 3.8	0.001
	3% NaOH	6% NaOH	– 2.7	– 4.6	– 1.0	0.008
		9% NaOH	– 6.7	– 9.3	– 3.7	0.001
	6% NaOH	9% NaOH	– 4.0	– 6.1	– 1.5	0.003
180 days	control	3% NaOH	– 2.1	– 3.2	– 0.6	0.002
		6% NaOH	– 1.9	– 4.4	0.2	0.106
		9% NaOH	– 1.6	– 3.5	0.1	0.092
	3% NaOH	6% NaOH	0.2	– 1.9	2.0	0.859
		9% NaOH	0.6	– 1.1	1.9	0.518
	6% NaOH	9% NaOH	0.3	– 1.9	2.8	0.775

a The mean difference is significant at a level of 0.05.

NaOH treatment may exert a stabilizing effect on deformation over time. Recent studies such emphasize that NaOH treatment of natural fibers enhances the interfacial adhesion of the fiber matrix, resulting in a more flexible surface conducive to integration into polymer composites.^67,68^ For instance, Maichin et al.,^39^ observed that treatment with higher concentrations of NaOH in hemp (*Cannabis sativa* L.) fibers led to increased interfacial adhesion between the fibers and the polymer matrix, thereby enhancing the mechanical properties of geopolymeric composites.

Similar to jute (*Corchorus olitorius* L.) and sisal (*Agave sisalana* P.) fibers, *Typha domingensis* contains cellulose as its primary component, contributing to favorable performance in terms of modulus of resistance to deformation and elasticity, given that *Typha* fiber typically contains around 85% cellulose.^69^ For instance, sisal fiber possesses a cellulose content of approximately 73%,^70^ while jute contains about 60% cellulose.^71^

Recent studies support these findings, such as those conducted by Pandey et al.,^25^ and Rahmawati et al.,^72^ which demonstrated that NaOH treatment significantly modifies the chemical composition and mechanical properties of *Typha domingensis* fibers. Pandey et al.,^25^ observed an increase in cellulose content following NaOH treatment, while Rahmawati et al.,^72^ confirmed the removal of amorphous components such as lignin and hemicellulose, leading to enhanced fiber crystallinity. This increase in crystallinity enhances stiffness but may also reduce fiber flexibility.

Fibers treated with higher concentrations of NaOH (6 and 9%) exhibited a decrease in tensile deformation over time compared to untreated fibers, indicating that higher alkalinity could increase stiffness while diminishing the fiber’s elongation capacity.^73^ Conversely, fibers treated with lower concentrations of NaOH (3%) demonstrated improved deformability over the 180-day exposure to natural degradation, aligning with recent studies indicating that moderate alkaline treatments enhance fiber flexibility.^42^

Hence, Figure 4 illustrates that *Typha domingensis* fibers treated with NaOH exhibit varying deformation behaviors depending on the concentration and biodegradation period. While higher NaOH concentrations lead to reduced deformability due to lignin degradation and increased stiffness, moderate concentrations (3% NaOH) provide a balance between tensile strength and flexibility, rendering them suitable for geotextile applications.^74^

Biodegradation of *Typha domingensis* fiber occurs as a result of enzymatic action by microorganisms, bacteria, and fungi naturally present in the soil.^75^ Unlike synthetic fibers and those of animal origin, which possess stabilized keratin compositions through intramolecular and intermolecular disulfide bonds, isopeptide bonds between amino groups of lysine and carboxyl groups of aspartic or glutamic acid, as well as numerous ionic, hydrogen bonds, and hydrophobic interactions, plant fibers have a distinct chemical structure influencing their biodegradability.^76,77^

The presence of disulfide bonds hinders access to peptide bonds by proteolytic enzymes. Under natural conditions, biodegradation typically occurs in two stages, requiring the synergistic action of various enzymes.^78,79^ In the initial stage, disulfide reductases break essential disulfide bonds, loosening the fiber’s compact structure and rendering it accessible to keratinases, which subsequently disrupt the peptide bonds.^78^

The disruption of disulfide and peptide bonds in plant fibers leads to the breakdown of the fiber structure and the degradation of long chains into shorter, water-soluble peptides, ultimately resulting in individual amino acid molecules.^80^ The molecular-level decomposition manifests as fiber discoloration, reduced mechanical strength, weight loss, and gradual fiber degradation.^81,82^

### Puncture Resistance

**Figure 5:**
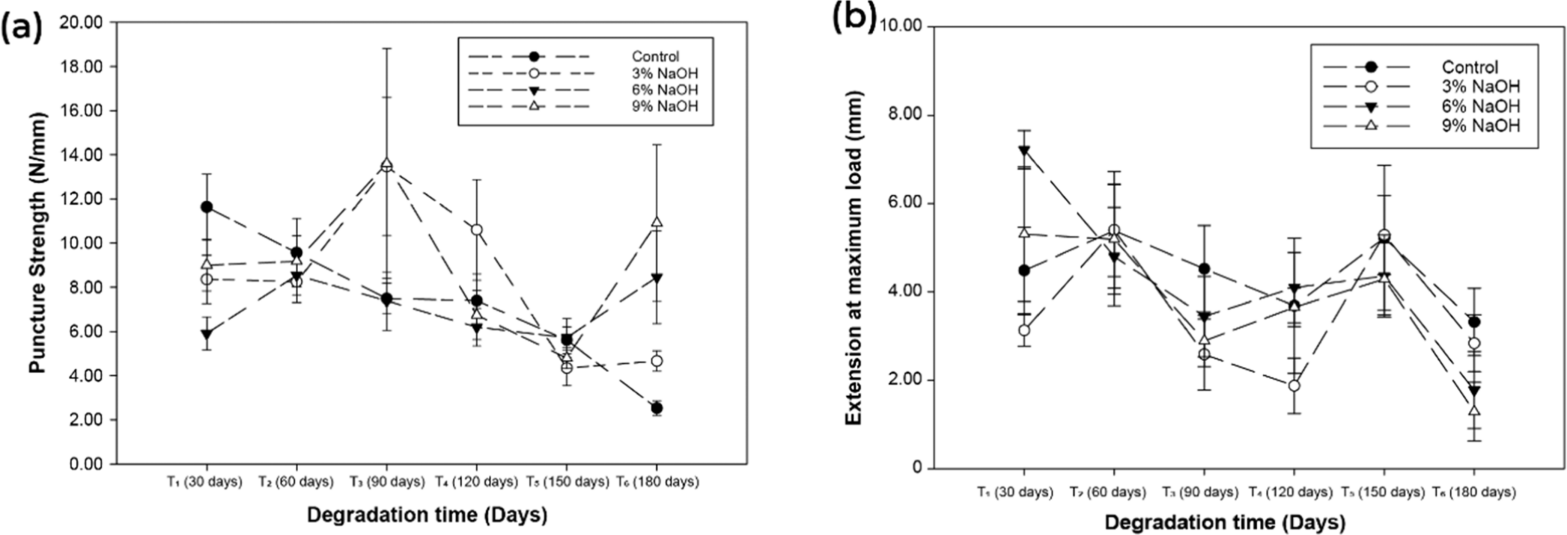
(a) Puncture resistance and (b) maximum extension of load of *Typha domingensis* fiber considering a field degradation time of 180 days.

Figure 5 illustrates the curve depicting the puncture resistance of *Typha domingensis* fibers as they are penetrated. Considering the static puncture strength of *Typha* treated with various concentrations of NaOH and exposed for different durations, the data in Figure 5a indicate that the puncture resistance of biodegradable *Typha domingensis* fibers fluctuates over time, contingent upon treatment and degradation duration, with additional influence from higher NaOH concentrations.

ANOVA analyses, incorporating different NaOH treatments and degradation times as factors, were conducted to elucidate the behavior of puncture resistance when subjected to sodium hydroxide treatment. The results of the corrected model (*p* < 0.001) signify that the factors included in the model significantly impact the puncture resistance of the geotextiles. The high value of the *Z* coefficient for the intercept (466.810) and its significance (*p* < 0.001) suggest that the baseline puncture resistance is statistically significant and well-defined, serving as a dependable reference point for treatment comparisons.

Regarding the NaOH treatments, the attributed variance was not statistically significant (*p* = 0.175), indicating a lack of discernible differences in puncture resistance solely due to NaOH treatments. However, the time factor exhibited a significant impact on puncture resistance (*p* = 0.001), with a squared partial η^2^ of 0.026. This implies that only 2.6% of the variability in puncture resistance can be attributed to differences between NaOH treatments, which is not statistically significant (*p* = 0.175). This behavior suggests that exposure to natural biodegradation over time primarily accounts for the decline in maximum puncture resistance, and NaOH treatment does not significantly affect material performance. This phenomenon may stem from the structural changes occurring in fibers during the biodegradation process.

The interaction between treatments and times was significant (*p* = 0.015), revealing that the effect of NaOH on puncture resistance is not constant over time, with the partial η^2^ being 0.137, which implies approximately 13.7%. This suggests that the effectiveness of NaOH treatment depends on the duration of exposure. For example, in certain periods of degradation, NaOH treatments strengthen the fibers to provide greater resistance, whereas in others they have a reduced effect due to continued degradation.

The bootstrap post hoc analysis of the dominance of the *Typha* fiber puncture resistance data, treated with NaOH and subjected to natural degradation in the field (Table 3), shows that, at the beginning of the experiment, after 30 days, the average difference between control and treatment with 3% NaOH is 3.277 N/mm, with a 95% confidence interval ranging from −0.267 to 7.148 N/mm, indicating that although there is a tendency for an increase in puncture resistance with NaOH treatment, this is not statistically significant (*p* = 0.096).

**Table 3:** Resistance to Fiber Puncture at the Beginning of the Experiment (30 Days) and after 180 Days of Exposure to Natural Degradation in the Field

				95% CI for mean difference		
exposure time	treatment	mean difference Δ*M* (I–J)		bottom	highest	sig. bonf
30 days	control	3% NaOH	3.277	–0.044	6.852	0.103
		6% NaOH	5.725^a^	2.722	9.008	0.006
		9% NaOH	2.626	–1.062	6.131	0.190
	3% NaOH	6% NaOH	2.448	–0.174	5.069	0.084
		9% NaOH	–0.651	–3.933	2.529	0.703
	6% NaOH	9% NaOH	–3.099^a^	–6.058	–0.187	0.047
180 days	control	3% NaOH	–2.130^a^	–3.250	–1.079	0.003
		6% NaOH	–5.924^a^	–11.272	–2.110	0.013
		9% NaOH	–9.030^a^	–17.462	–2.020	0.039
	3% NaOH	6% NaOH	–3.793	–9.269	0.123	0.099
		9% NaOH	–6.900	–15.003	0.204	0.127
	6% NaOH	9% NaOH	–3.107	–13.183	5.298	0.524

a The mean difference is significant at a level of 0.05.

Treatment with 6% NaOH shows a more remarkable increase (mean difference 5.725 N/mm, *p* = 0.005), suggesting potential fiber strengthening. However, the 9% NaOH treatment does not show a significant increase in puncture resistance compared to the control (mean difference of 2.626 N/mm, *p* = 0.199). At 60 days, the differences become less pronounced, with the 3% NaOH treatment showing a small mean difference of 1.323 N/mm and a 95% confidence interval that does not exclude the zero value (−1.566 to 4.596 N/mm), which implies a variation in puncture resistance that could be due to chance (*p* = 0.480).

NaOH treatments affect puncture resistance in a variable way over time. Specifically, treatment with 3% NaOH shows a decrease in puncture resistance compared to control (mean difference of −2.130 N/mm, *p* = 0.004), suggesting a possible long-term fiber vulnerability. The results indicate that the puncture resistance of *Typha domingensis* fiber can be beneficially influenced by NaOH treatment, especially at a concentration of 9% considering the 180 days of exposure to biodegradation.

These findings align with recent studies indicating enhanced interfacial adhesion of the fiber matrix following NaOH treatment, potentially leading to a more flexible surface and improved integration into polymer composites. For instance, Nwaiwu et al.,^83^ highlighted that coconut (*Cocos nucifera*) fibers treated with 15% NaOH exhibited increased extensibility and resistance to perforation. Additionally, Saavedra et al.,^84^ demonstrated that NaOH treatment reduced the hydrophilic nature of buriti palm (*Mauritius flexuosa*) fibers, enhancing their compatibility with hydrophobic matrices and potentially improving puncture resistance.

Regarding the extension data at maximum load depicted in Figure 5b, which typically refers to fiber deformation before reaching its maximum load capacity (the point just before breaking), it is evident that after 30 days, the 3% NaOH treatment did not exhibit significant differences compared to the control (mean difference of 1.329 mm, *p* = 0.051), suggesting minimal changes in fiber flexibility.

However, after 180 days, a noticeable increase was observed in the difference in mean extension at maximum load for fibers treated with 6% and 9% NaOH (Δ*M* of 1.395 and 2.277 mm, respectively with, *p* = 0.066 and *p* < 0.001). This indicates that structural modifications resulting from high concentrations of NaOH affect the extensibility and flexibility of the fibers. This observation is supported by the fact that, concurrently, the maximum extension of treated samples decreases with exposure to degradation while the control sample remains stable (Figure 5b), suggesting that NaOH treatments can impact fiber elasticity without necessarily compromising puncture resistance.

This tendency can have both beneficial and detrimental implications, depending on the intended application of the fibers. For instance, in scenarios where elasticity is favored over puncture resistance, such as in woven textiles, higher concentrations of NaOH and longer degradation periods may prove advantageous. Conversely, in applications where puncture resistance is paramount, such as in geotextiles, this trade-off warrants careful consideration. These findings are consistent with those of,^85^ who observed that increased NaOH concentrations enhance interfacial adhesion between Juncus (*Eleocharis* spp.) fibers and polymer matrices, resulting in improved mechanical performance, including extensibility, in geopolymeric composites.

## Conclusions

NaOH treatment has been shown to positively impact fiber tensile strength, particularly at a concentration of 9% (3.61 N/mm^2^) with a maximum load capacity of 25.55 N, indicating enhanced fiber durability. However, over a 180-day biodegradation period, treated fibers exhibited a decline in puncture resistance, indicating potential long-term structural fragility. Nonetheless, this treatment improved interfacial adhesion to the fiber matrix, potentially contributing to enhanced mechanical properties in polymer composites.

The ductility and flexibility of fibers vary depending on NaOH concentration and exposure time, with implications that can be advantageous or detrimental based on the specific application. For scenarios necessitating puncture resistance, such as in geotextiles for erosion control, higher NaOH concentrations ranging between 6% and 9% are recommended. Conversely, when elasticity is desired but puncture resistance is not critical, it is essential to adhere to the minimum concentration limits established by this research, which range between 6 and 3% NaOH concentration.

The durability of NaOH-treated fibers underscores that while natural fibers may biodegrade more rapidly, proper treatment can extend their service life, rendering them a sustainable alternative to traditional synthetic materials for up to 180 days. Treatment with NaOH in *Typha domingensis* fiber presents a promising approach for enhancing the mechanical properties of natural fibers in geotextile applications, thereby contributing to soil bioengineering practices controlling erosion in an environmentally sustainable manner.
